# Tailoring limb length based on total small bowel length in one anastomosis gastric bypass surgery (TAILOR study): study protocol for a randomized controlled trial

**DOI:** 10.1186/s13063-022-06456-w

**Published:** 2022-06-22

**Authors:** Nienke Slagter, Loek J. M. de Heide, Ewoud H. Jutte, Mirjam A. Kaijser, Stefan L. Damen, André P. van Beek, Marloes Emous

**Affiliations:** 1 Center for Obesity Northern Netherlands (CON), Medical Center Leeuwarden, Henri Dunantweg 2, 8934 AD Leeuwarden, The Netherlands; 2 University of Groningen, University Medical Center Groningen, Groningen, The Netherlands; 3 Department of Endocrinology, University of Groningen, University Medical Center Groningen, Groningen, the Netherlands

**Keywords:** Bariatric surgery, One anastomosis gastric bypass, Biliopancreatic limb, Tailoring limb length, Total small bowel length

## Abstract

**Background:**

The one anastomosis gastric bypass (OAGB) is being performed by an increasing number of bariatric centers over the world. However, the optimal length of the biliopancreatic (BP) limb remains a topic of discussion. Retrospective studies suggest the benefit of tailoring BP-limb length; however, randomized trials are lacking. The aim of this study is to investigate whether tailoring the length of the BP-limb based on total small bowel length (TSBL) leads to better results in terms of weight loss, vitamin deficiencies, and bowel movements compared to a fixed BP-limb length.

**Methods:**

The TAILOR study is a double-blind single-center randomized controlled trial. Patients scheduled for primary OAGB surgery will be randomly allocated either to a standard BP-limb of 150 cm or to a BP-limb length based on their TSBL: TSBL < 500 cm, BP-limb 150 cm; TSBL 500–700 cm, BP-limb 180 cm; TSBL > 700 cm, BP-limb 210 cm. The primary outcome is to compare the percent total weight loss (%TWL) at 5 years between the two groups. Secondary outcomes include nutritional deficiencies, remission of comorbidities, symptoms of dumping, quality of life, and daily bowel movements. The study includes a total of 212 patients and is designed to detect a 5% difference in the primary endpoint.

**Discussion:**

The TAILOR study will provide new insights into the effect of different BP-limb lengths and the role of the TSBL in the OAGB. The study is designed to provide guidance for bariatric surgeons to determine the optimal BP-limb length in the OAGB.

**Trial registration:**

Dutch Trial Register NL7945. Prospectively registered on 08 September 2019. NTR (trialregister.nl)

**Supplementary Information:**

The online version contains supplementary material available at 10.1186/s13063-022-06456-w.

## Administrative information

**Table:** 

**Trial registration**	Dutch trial register: NL7945
**Date registration**	09-08-2019
**Trial status**	We started participant recruitment in September 2020 and we expect to complete the recruitment in November 2022.
**Version**	2
**Sponsor**	Medical Center Leeuwarden 8934 AD Leeuwarden The Netherlands
	Phone: +31 58 286 6969
**Principal investigator(s)**	L.J.M. de Heide, MD, Internist-Endocrinologist, Center for Obesity Northern Netherlands (CON)/Medical Center Leeuwarden
**Roles and responsibilities**	Daily support and coordination of the study is provided by: *Principle investigator*: supervision of the study, annual safety reports, and trial registration. *Study coordinator*: coordinates study, ensures follow-up according to protocol, reporting Serious Adverse Events (SAE), takes informed consent, organizes data capture and collection, and follow-up of study patients. *Surgeons:* identifies potential recruits, explanation of the study, and applying the intervention. *Study physician/physician assistant*: explanation of the study, takes informed consent, data collection, and follow-up of study patients.

## Introduction

The one anastomosis gastric bypass (OAGB) is currently one of the most effective treatment options for morbid obesity. It is a technically simpler procedure compared to the gold standard, the Roux-en-Y gastric bypass (RYGB), and has proven to have equivalent outcomes in terms of weight loss and reduction of comorbidities [1–3]. Currently, there is no standard guideline for the optimal biliopancreatic (BP) limb length for the OAGB. The aim of a suitable BP-limb is to achieve optimum weight loss in combination with a minimum of side effects as nutritional deficiencies and diarrhea [4, 5]. There is substantial variation in the determination of the BP-limb length, as some surgeons use a fixed length and some tailor the length based on body mass index (BMI), age, gender, or comorbidities [1, 2, 4, 6–8]. Furthermore, the lengths of the applied BP-limb vary between less than 150 cm and more than 250 cm.

Evidence exists in RYGB surgery that the length of the BP-limb influences weight loss. Nergaard et al. performed a randomized controlled trial (RCT) comparing a RYGB with a BP-limb of 200 cm with an alimentary limb of 60 cm to a RYGB with a BP-limb of 60 cm with an alimentary limb of 150 cm. The longer BP-limb length led not only to more weight loss, but also to more bowel movements and micronutrient deficiencies [9]. Zorrilla et al. performed a systematic review on limb length in RYGB and found weight loss on the whole was better in patients with longer BP-limbs [10].

Compared to the RYGB, there is less research about the effect of different limb lengths in the OAGB. A study of Ahuja et al. adjusted the BP-limb length in OAGB from 150 to 250 cm depending on age, sex, BMI, comorbidities, and diet. They found an increasing number of deficiencies of micronutrients with a BP-limb length of 250 cm [5]. In our own retrospective study, we found that tailoring BP-limb length based on baseline BMI did not abrogate the BMI differences at 3 years with also no differences in the resolution of comorbidities [11].

The total length of the small bowel can vary considerably with measured values between 350 and more than 1000 cm. In a study of 443 patients, the median total small bowel length (TSBL) was 690 cm with a wide SD of 94 cm [12]. The effect of a certain BP-limb length may be influenced by the high variance in TSBL. Two recent retrospective studies showed promising results of less nutritional deficiencies when adjusting the BP-limb based on the TSBL [13, 14]. These retrospective studies emphasize the need for prospective randomized studies.

The aim of this RCT is to investigate if tailoring the BP-limb length based on TSBL is superior compared to a fixed BP-limb length in terms of weight loss, nutritional deficiencies, bowel movements, and resolution of comorbidities.

## Methods

### Study design

This is a double-blind single-center RCT with two arms and a follow-up period of 5 years. The study is being conducted in a non-academic teaching hospital in the Northern Netherlands. Patients are eligible to participate if they are scheduled for primary OAGB surgery in the Center for Obesity Northern Netherlands (CON) and are willing to participate. All patients comply with the International Federation for the Surgery of Obesity and Metabolic Disorders (IFSO) guidelines and undergo a multidisciplinary screening. The enrolment of patients started in July 2020 and the first patients underwent surgery in September 2020.

### Study population

#### Inclusion criteria

In order to be eligible to participate in this study, a subject must meet the following criteria:

- Scheduled for primary OAGB
- Age between 18 and 65 years
- BMI > 40 kg/m^2^ or BMI > 35 kg/m^2^ with comorbidity: diabetes mellitus type 2 (T2D), hypertension, obstructive sleep apnea (OSA), cardiac disease, or arthrosis
- No preoperative deficiencies of vitamin B_12_, D, and iron (measured as ferritin)
- No use of extra (multi-)vitamin supplements with exception of vitamin D max 800 IU/day
- Willing to participate with written informed consent before the start of the surgery
- Able to swallow the multivitamin Fit For Me (FFM) weight loss surgery (WLS) primo (tested before surgery)
- A completely measured TSBL during laparoscopic surgery

#### Exclusion criteria

A potential subject who meets any of the following criteria will be excluded from participation in this study:

- BMI > 50 kg/m^2^
- Known gastro-intestinal disease or history of gastro-intestinal disease, e.g., celiac disease and inflammatory bowel disease
- Known addiction behavior
- Intolerance to FFM WLS primo multivitamin
- Pregnancy planning within the first 2 years after surgery
- Renal or hepatic insufficiency
- Former abdominal surgery and therefore not able to measure the small bowel length during laparoscopic surgery
- Parenteral use of vitamin B_12_

### Intervention

During the surgery, patients will be allocated to one of the two surgical treatment arms after complete measurement of the TSBL. Eligible patients will be randomized in equal proportions in both groups.

1. A standard BP-limb length of 150 cm
2. A BP-limb length depending on TSBL measured during the OAGB procedure:
  TSBL < 500 cm: BP-limb 150 cm
  TSBL 500–700 cm: BP-limb 180 cm
  TSBL > 700 cm: BP-limb 210 cm

Patients with a TSBL that could not be measured during surgery will be treated according to the current daily practice and will not enter the study. Currently, in our bariatric center, a fixed BP-limb length of 150 cm is performed in daily practice.

#### Non-investigational product

After bariatric surgery, a lifelong daily intake of WLS multivitamin is recommended. In order to make vitamin supplementation uniform during the study, patients will be provided with daily multivitamin FFM WSL primo during the 5-year follow-up. FFM WSL primo is a multivitamin and mineral containing product with a content that is especially developed for patients after OAGB.

### Study parameters

#### Primary outcome

To compare the percent total weight loss (%TWL) at 5 years between the group with the standard BP-limb length and the group with an adjusted BP-limb length.

#### Secondary outcome

As shown in Table 1, secondary outcomes are %TWL at the other follow-up moments; percent excess weight loss (%EWL); percent excess BMI loss (%EBMIL); proportion of patients with 22 ≤ BMI ≤ 30 kg/m^2^; daily bowel movements; total and partial remission of T2D, hypertension, and OSA; nutritional deficiencies; quality of life measured by the OBESI-Q questionnaire [15]; and percentage of patients experiencing dumping symptoms defined by the Dumping Severity Score (DSS) [16]. All secondary outcome parameters will be compared between the groups with the standard BP-limb length and the adjusted BP-limb length. Furthermore, analysis in the subgroups with different TSBL will be performed. Total remission of T2D is defined as an HbA1c less than 48 mmol/mol without diabetes medication in the last 6 months. Improvement of diabetes is defined as a reduction of HbA1c of 10 mmol/mol or more but not reaching remission criteria and/or less anti-diabetic medication. The definition of total remission of hypertension is being able to stop all antihypertensive medication. Improvement of hypertension is defined as less antihypertensive medication. Resolution of OSA is defined by cessation of continuous positive airway pressure (CPAP) or other device use, documented by their own pulmonologist.

**Table 1:** Outcome parameters

	Outcome	Measurement	Time point
**Primary**	Percent total weight loss	%TWL	5 years
**Secondary**	Percent total weight loss	%TWL	Other follow-up moments
	Weight loss	• %EWL • %EBMIL • 22 ≤ BMI ≤ 30 kg/m^2^	Each follow-up moment
	Defecation	• Mean daily bowel movements • Number of days with daily bowel movements > 3 in the last 2 weeks	Each follow-up moment
	Remission of T2D, hypertension, and OSA	• Current use of medication • HbA1c • Blood pressure • Use of CPAP or other devices	Each follow-up moment
	Nutritional deficiencies	Vitamins A, B_1_, B_6_, B_12_, and D; iron; folic acid; calcium; phosphate; albumin; and zinc	6 months and 1, 2, 3, 4, and 5 years
		Copper and selenium	1, 3, and 5 years
	Quality of life	OBESI-Q [15]	Preoperative, 6 months, 12, 18, 30, 42, 54, and 60 months
	Symptoms of dumping syndrome	DSS [16]	Preoperative, 6 months, 12, 18, 30, 42, 54, and 60 months

*TWL* total weight loss, *EWL* excess weight loss, *EBMIL* excess BMI loss, *BMI* body mass index, *T2D* diabetes mellitus type 2, *OSA* obstructive sleep apnea

Follow-up moments: 6, 12, 18, 24, 30, 36, 42, 48, 54, and 60 months

#### Other study parameters

Measurements of CRP, hemoglobin, WBC, thrombocytes, sodium, potassium, creatinine, BUN, ASAT, ALAT, alkaline phosphatase, yGT, and HbA1c are part of the routine investigation in the follow-up and will also be part of the study. The serum will be stored for analysis in the later stage in case of new developments or insights relevant for the study become evident.

### Study procedure

#### Recruitment

Patients will be asked to participate by the bariatric surgeon during their preoperative visit. After a verbal explanation of the study, they will be provided with written patient information and a consent form. After 2–4 weeks, they will be approached by a physician or a physician assistant of the research team. Any questions still not answered will be addressed and patients will be asked to sign the informed consent form. The patient will be asked to swallow one FFM WLS primo capsule in order to see whether they are able to swallow the capsule.

#### Randomization, blinding, and treatment allocation

**Fig. 1:**
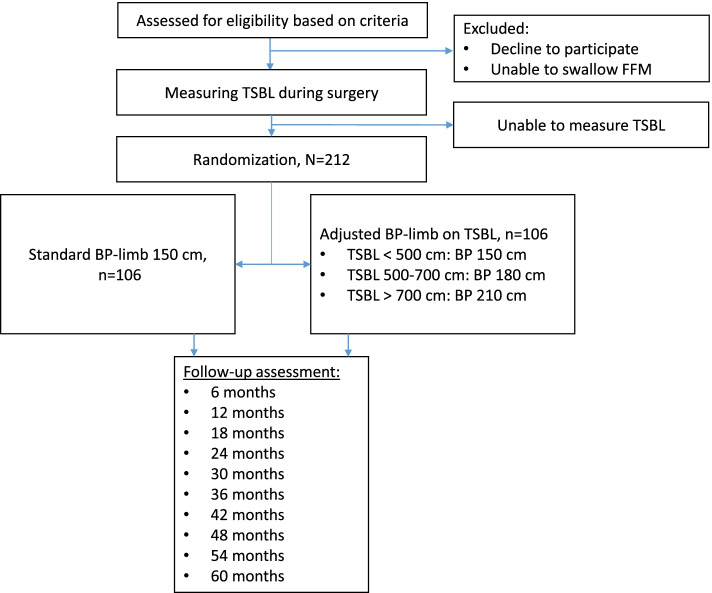
Flowchart describing the timeline of the study

Participants will be randomly assigned to either the control or the experimental group with a simple randomization with 1:1 allocation (Fig. 1). Randomization is performed using a table of random figures delivered by an independent party (clinical pharmacist), using sealed envelopes with the treatment allocation. The sealed envelope will be opened by the surgery assisting nurse after the surgeon has succeeded in measuring the TSBL. Only the operating surgeon is aware of the allocation.

An employee of the CON outside the research team will document TSBL, treatment allocation, and BP-limb length in a secured coded database containing only study numbers. The length of the BP-limb and treatment allocation will not be documented in the patient file, and the research team, other CON care providers, and the patient will not be informed.

#### Preoperative assessment and surgery

Patients will be asked to fill in the OBESI-Q and DSS questionnaires before the surgery. A standard operation protocol for OAGB will be used in all patients and applied by four skilled bariatric surgeons. The surgical technique has been described before by Apers et al. [17]. After the creation of the gastric pouch, the TSBL is measured. The TSBL is measured performing the stepwise hand-over-hand technique using two marked graspers to pass the small bowel in estimated steps of 5 cm. This is the same technique performed in daily practice to measure BP-limb length. Based on the randomization outcome, the standard or adjusted BP-limb length is applied. After the surgery, baseline data of the patients as preoperative weight, blood pressure, comorbidities, perioperative data, and complications will be recorded in the database. On leave of the hospital, the patients will be provided with FFM WSL primo multivitamin capsules for the first half year. All patients will also be treated with 800 IU vitamin D and 500 mg calcium as part of standard care.

#### Follow-up assessment

Follow-up will take place in accordance with usual care, i.e., after 6 and 18 months and yearly up till 5 years. Extra visits will be at 30, 42, and 54 months. At each follow-up visit, standard care includes inquiry after well-being and complaints, daily bowel movements, current medication use, evaluation of comorbidities, and measurement of body weight, blood pressure, and pulse. Patients will be asked to fill in the OBESI-Q and DSS questionnaires before the follow-up appointment. For counting of compliance, patients will be asked to bring back all used and non-used blisters of the FFM WLS primo multivitamins. Blood samples will be taken in accordance with the standard protocol of follow-up after bariatric surgery, added with 20 ml extra blood draw for selenium and copper measurements at 1-, 3-, and 5-year visits. These samples will be stored in a freezer at a laboratory in the hospital and will be used in the current study and also in the future for ancillary studies. In case of deficiencies of vitamins or micronutrients, they will be substituted according to standard care. Patients receive a package with 6 months of FFM multivitamins at each follow-up visit to improve adherence to follow-up protocol.

#### Potential harm

Measuring TSBL induces a slight increase in the risk of damaging the bowel compared to the standard procedure of solely measuring the length of the BP-limb. In addition, this measurement will increase the time of surgery with on average 10 min.

In OAGB surgery, surgeons use BP-limb lengths varying between 150 and 250 cm based on personal experience. The different lengths in our study are within these measures. Potential risks are too much or too little weight loss and more than expected deficiencies of vitamins and micronutrients. Potential benefits are a better outcome of weight reduction compared to current usual care without an increase in vitamin and/or micronutrient deficiencies.

### Statistics and data management

#### Sample size calculation

The hypothesis of the study is that the mean %TWL in the experimental group is 5% more than the standard group (40% and 35%, respectively). A sample size of 2 × 92 will achieve 80% power to detect a difference of 5% with estimated group standard deviations of 12 and with a significance level (alpha) of 0.05. With an expected maximum drop-out due to conversion of OAGB to RYGB of 15%, the sample size is 106 patients in each group, leading to a total of 212 participants in the study.

#### Data management

Data will be recorded on an electronic case report form (CRF) using Gemstracker (Generic Medical Survey Tracker, Erasmus MC Rotterdam). The questionnaires of OBESI-Q and DSS will be sent automatically to the patients by email using the questionnaire function of LimeSurvey (LimeSurvey Project, Hamburg, Germany) in Gemstracker. An automatic reminder will be sent after 7 days. Extracting the data from Gemstracker, data will be coded using a unique numerical code. Data concerning participants will be kept confidential and will only be accessible for study team members. Treatment allocation will be recorded on a password secured hard drive in a database containing only study numbers. Any data required to support the protocol can be supplied on request.

#### Statistical analysis

The full analysis set will include all randomized patients with at least one post-baseline measurement in accordance with the intention-to-treat principle. The primary outcome and continuous secondary outcomes of the two treatment strategy groups will be compared using a two-sample *T*-test or Mann-Whitney *U*-test, for normal (mean ± SD) or skewedly distributed (median [IQR]) data, respectively. For categorical secondary outcomes, a Fisher exact test or a chi-square test will be used. In addition to the abovementioned analyses, a multivariable model will be used to formally correct for the effect modification of potentially relevant factors on the treatment effect. Missing data will remain as missing, and no attempt will be made to estimate or replace missing values. A *p*-value of ≤ 0.05 will be considered to indicate statistical significance.

#### Monitoring and quality assurance

Based on the current experience of the surgeons with the procedures, a data safety monitoring board (DSMB) is not considered necessary. An independent physician with experience in trials will be asked to monitor the quality of data acquisition and documentation. The principal investigator will report all serious adverse events (SAE) to the sponsor after obtaining knowledge of the events without undue delay. All adverse events (AE) reported spontaneously by the subject or observed by the research team members will be recorded. The deblinding of the allocated treatment will be performed by an independent person, who is not part of the investigation team. Reasons for premature termination of the study are an unexpectedly high rate of surgical complications due to measuring TSBL or adjusting the BP-limb length as to the discretion of the investigator. Interim analyses will be performed after all patients have completed the visits at 1, 2, 3, and 4 years. A data safety monitoring board was not instituted according to Dutch legislation as this is expected to be a low-risk study.

#### Withdrawal

Patients who develop complications needing revisional surgery during the first 30 days after the surgery will be withdrawn and replaced. Patients who are not able to tolerate the FFM WSL primo capsule after the surgery, patients who need a conversion from the OAGB to a RYGB, and patients not meeting study compliance will be withdrawn from the study and will not be replaced. They will be analyzed on an intention-to-treat principle and all data up to the exclusion will be used in the study.

### Ethics and dissemination

#### Ethics committee

Ethical approval has been obtained from the medical ethical committee (METC): RTPO, Leeuwarden (NR 1082). In addition, the principal investigator will submit a summary of the progress of the trial to the accredited METC once a year. In the case of any modifications of the study protocol that may impact the conduct of the study, the medical ethical committee will be notified. In accordance with the legal requirement in the Netherlands, the sponsor has an insurance to compensate for injury during and after the study (Article 7 Medical Research Involving Human Subjects Act (WMO).

#### Public disclosure and publication policy

The results of the study will be published in a peer-reviewed medical journal after agreement of all participating authors. FFM will not be involved in the decision to publish nor in the content of the publication. The Central Committee on Research Involving Human Subjects (CCMO) statement concerning publication policy will be followed.

## Discussion

The TAILOR study is the first RCT to investigate the effect of tailoring limb length based on TSBL compared to a fixed BP-limb length in terms of weight loss, nutritional deficiencies, and resolution of comorbidities. This study is designed to provide guidance for bariatric surgeons to determine the optimal BP-limb length in the OAGB.

The available literature exists on retrospective studies with subjective variations, limiting the possibility to compare studies and therefore hinder consensus on the optimal BP-limb length. Furthermore, the impact of the size of the TSBL on bypassing a certain part of the small bowel remains unclear. A short TSBL may result in a short residual bowel with the risk of serious nutritional deficiencies and a long TSBL might result in a long residual bowel with the consequence of poor weight loss. We expect that the outcomes of the TAILOR study will provide knowledge about the effect of different BP-limb lengths and the role of the TSBL in the OAGB. We hypothesize that adjusting the length of the BP-limb based on measured TSBL leads to more weight loss with less development of micronutrient deficiencies and bowel movements compared to a fixed limb length.

This study has some limitations. First, the TSBL is measured using a stepwise hand-over-hand technique using marked graspers to pass the small bowel in estimated steps of 5 cm. As the steps are estimated by the bariatric surgeon, this is not an objective measurement technique. Therefore, it probably results in the variation of the measured lengths of the BP-limb and TSBL. However, this is currently the daily practice. Furthermore, the applied BP-limb lengths of 150, 180, and 210 cm may show little differences, where larger differences might provide more clarity of the effect. Nevertheless, some studies found increasing numbers of micronutrient deficiencies with a BP-limb length of 250 cm, and therefore, we choose all BP-limb lengths below the 250 cm [5].

## Supplementary Information

**Additional file 1.** Informed consent

## Data Availability

The datasets analyzed during the current study and statistical code are available from the corresponding author on reasonable request, as is the full protocol.

## Abbreviations

AE: Adverse event
BMI: Body mass index
BP: Biliopancreatic
CCMO: Central Committee on Research Involving Human Subjects
CON: Center for Obesity Northern Netherlands
CPAP: Continuous positive airway pressure
CRF: Case report form
DSMB: Data safety monitoring board
DSS: Dumping Severity Score
EBMIL: Excess BMI loss
EWL: Excess weight loss
FFM: Fit For Me
IFSO: International Federation for the Surgery of Obesity and Metabolic Disorders
METC: Medical ethical committee
OAGB: One anastomosis gastric bypass
OSA: Obstructive sleep apnea
RCT: Randomized controlled trial
RYGB: Roux-en-Y gastric bypass
SAE: Serious adverse events
T2D: Diabetes mellitus type 2
TSBL: Total small bowel length
TWL: Total weight loss
WLS: Weight loss surgery
%TWL: (Initial weight − postoperative weight)/initial weight × 100
%EWL: (Initial weight − postoperative weight)/(initial weight − ideal weight) × 100
Ideal weight: Weight corresponding to a BMI of 25 kg/m^2^
%EBMIL: ΔBMI/(initial BMI − 25) × 100
