# Supplementary material for: Tailoring limb length based on total small bowel length in one anastomosis gastric bypass surgery (TAILOR study): study protocol for a randomized controlled trial
Source: Trials. 2022 Jun 22;23:526. doi: 10.1186/s13063-022-06456-w (PMC9214974; doi:10.1186/s13063-022-06456-w)
Supplement: Supplementary file 1 — Additional file 1. Informed consent [file 13063_2022_6456_MOESM1_ESM.docx]

Bijlage C: Toestemmingformulier proefpersoon de TAILOR studie

- Ik heb de informatiebrief gelezen. Ook kon ik vragen stellen. Mijn vragen zijn voldoende beantwoord. Ik had genoeg tijd om te beslissen of ik meedoe.
- Ik weet dat meedoen vrijwillig is. Ook weet ik dat ik op ieder moment kan beslissen om toch niet mee te doen of te stoppen met het onderzoek. Daarvoor hoef ik geen reden te geven.
- Ik geef toestemming voor het informeren van mijn huisarts dat ik meedoe aan dit onderzoek.
- Ik geef toestemming voor het verzamelen en gebruiken van mijn gegevens en voor de beantwoording van de onderzoeksvraag in dit onderzoek.
- Ik weet dat voor de controle van het onderzoek sommige mensen toegang tot al mijn gegevens kunnen krijgen. Die mensen staan vermeld in deze informatiebrief. Ik geef toestemming voor die inzage door deze personen.
- Ik geef toestemming voor het informeren van mijn huisarts en/of behandelend specialist van onverwachte bevindingen die van belang (kunnen) zijn voor mijn gezondheid.
- Ik geef □ **wel** □**geen**
  toestemming om mijn persoonsgegevens en lichaamsmateriaal langer te bewaren en te gebruiken voor toekomstig onderzoek op het gebied van obesitas.
- Ik geef □ **wel** □**geen**toestemming om bij mijn apotheek informatie op te vragen tijdens de studie over afgeleverde medicatie.
- Ik geef □ **wel** □**geen**toestemming om de gegevens, die tijdens het onderzoek verkregen zijn, geanonimiseerd ter beschikking te stellen aan FitForMe.
- Ik geef □ **wel** □**geen**toestemming om mij na dit onderzoek opnieuw te benaderen voor een vervolgonderzoek.
- Ik wil meedoen aan dit onderzoek.

**Naam proefpersoon:**

**Geboortedatum:**

Handtekening: Datum : __ / __ / __

-----------------------------------------------------------------------------------------------------------------

Ik verklaar dat ik deze proefpersoon volledig heb geïnformeerd over het genoemde onderzoek.

Als er tijdens het onderzoek informatie bekend wordt die de toestemming van de proefpersoon zou kunnen beïnvloeden, dan breng ik hem/haar daarvan tijdig op de hoogte.

**Naam onderzoeker (of diens vertegenwoordiger):**

Handtekening: Datum: __ / __ / __

-----------------------------------------------------------------------------------------------------------------
